# PACS-2 deficiency in tubular cells aggravates lipid-related kidney injury in diabetic kidney disease

**DOI:** 10.1186/s10020-022-00545-x

**Published:** 2022-09-23

**Authors:** Chanyue Zhao, Li Li, Chenrui Li, Chengyuan Tang, Juan Cai, Yu Liu, Jinfei Yang, Yiyun Xi, Ming Yang, Na Jiang, Yachun Han, Yan Liu, Shilu Luo, Li Xiao, Lin Sun

**Affiliations:** Department of Nephrology, Hunan Key Laboratory of Kidney Disease and Blood Purification, The Second Xiangya Hospital, Central South University, No.139 Renmin Middle Road, Changsha, 410011 Hunan China

**Keywords:** Diabetic kidney disease, PACS-2, SOAT1, SREBP

## Abstract

**Background:**

Lipid accumulation in tubular cells plays a key role in diabetic kidney disease (DKD). Targeting lipid metabolism disorders has clinical value in delaying the progression of DKD, but the precise mechanism by which molecules mediate lipid-related kidney injury remains unclear. Phosphofurin acidic cluster sorting protein 2 (PACS-2) is a multifunctional sorting protein that plays a role in lipid metabolism. This study determined the role of PACS-2 in lipid-related kidney injury in DKD.

**Methods:**

Diabetes was induced by a high-fat diet combined with intraperitoneal injections of streptozotocin (HFD/STZ) in proximal tubule-specific knockout of *Pacs-2* mice (*PT*-*Pacs-2*^*−/−*^ mice) and the control mice (*Pacs-2*^fl/fl^ mice). Transcriptomic analysis was performed between *Pacs-2*^fl/fl^ mice and *PT*-*Pacs-2*^*−/−*^ mice.

**Results:**

Diabetic *PT*-*Pacs-2*^*−/−*^ mice developed more severe tubule injury and proteinuria compared to diabetic *Pacs-2*^fl/fl^ mice, which accompanied with increasing lipid synthesis, uptake and decreasing cholesterol efflux as well as lipid accumulation in tubules of the kidney. Furthermore, transcriptome analysis showed that the mRNA level of sterol *O*-acyltransferase 1 (*Soat1*) was up-regulated in the kidney of control *PT*-*Pacs-2*^*−/−*^* mice*. Transfection of HK2 cells with PACS-2 siRNA under high glucose plus palmitic acid (HGPA) condition aggravated lipid deposition and increased the expression of SOAT1 and sterol regulatory element-binding proteins (SREBPs), while the effect was blocked partially in that of co-transfection of SOAT1 siRNA.

**Conclusions:**

PACS-2 has a protective role against lipid-related kidney injury in DKD through SOAT1/SREBPs signaling.

**Supplementary Information:**

The online version contains supplementary material available at 10.1186/s10020-022-00545-x.

## Background

Diabetic kidney disease (DKD) is chronic kidney disease caused by diabetes, and is highly prevalent across the globe (Hussain et al. 2021). The main histological features of the disease are glomerular and tubular basement membrane thickening, mesangial expansion and mesangiolysis, arteriolar hyalinosis and tubulointerstitial fibrosis (Thomas 2021). Recently there is clear evidence showing that tubular injury plays a pivotal role in the pathogenesis of DKD (Vallon 2011), and it is superior to glomerular pathology as a predictor of CKD progression (Bohle et al. 1987; Risdon et al. 1968). The main mechanisms of tubular injury in DKD include a series of injury pathways caused by hyperglycemia, oxygen metabolism disorders, inflammation, fibrosis and apoptosis (Duan et al. 2021), but new molecules related to tubular injury in DKD still need to be identified.

Lipid accumulation in tubular cells is a common phenomenon in patients with DKD (Chen et al. 2019; Herman-Edelstein et al. 2014). Proximal tubular cells have high levels of baseline energy consumption and fatty acids are the preferred substrate for proximal tubule ATP generation under normal circumstances (Guder et al. 1986; Nieth and Schollmeyer 1966). Therefore, proximal tubule cells are more susceptible to lipid metabolism disorders. Moorhead et al. (1982) first proposed that lipid accumulation contributes to chronic kidney injury in 1982, increasing evidence supports that excess lipid in kidney could lead to a series of kidney injuries by endoplasmic reticulum (ER) stress, mitochondrial dysfunction, reactive oxygen species (ROS) production, cell apoptosis, etc. (Declèves et al. 2014; Hosokawa et al. 2019; Khan et al. 2014; Weinberg 2006; Wu et al. 2021c). Studies by our group and others have shown that renal lipid accumulation is positively correlated with the tubulointerstitial damage in DKD (Chen et al. 2019; Han et al. 2021; Yang et al. 2018). Proposed mechanisms leading to lipid accumulation in proximal tubule include increased uptake of lipid, increased synthesis of lipid, diminished β-oxidation and cholesterol efflux (Kang et al. 2015; Proctor et al. 2006; Sun et al. 2002). Our previous studies also show that disulfide-bond A oxidoreductase-like protein protects against lipid-related kidney damage in DKD by increasing triglyceride hydrolysis and decreasing cholesterol synthesis (Chen et al. 2019) and identify that the deficiency of lipophagy, a process by which lipid droplets are degraded via autophagy, also plays an important role in ectopic lipid accumulation in DKD (Han et al. 2021). However, other molecules related to lipid-related kidney injury still need to be further identified.

Phosphofurin acidic cluster sorting protein 2 (PACS-2) is a member of PACS family expressed in skeletal muscle, brain, heart and kidney, and it plays important roles in membrane trafficking, autophagy and apoptosis (Li et al. 2020). Recently, the role of PACS-2 in lipid metabolism has attracted attention. Simmen et al. (2005) suggested that PACS-2 controlled the formation of ER lipid-synthesizing centers found at mitochondria-associated endoplasmic reticulum membranes (MAMs) and the knockdown of PACS-2 significantly reduced the amount of long chain fatty acid CoA ligase 4 (FACL4) and phosphatidylserine synthase 1 (PSS1) in the MAM fraction. Besides, *Pacs-2* knockout mice have elevated liver sirtuin 1 (SIRT1) activity and are protected from diet-induced obesity (Krzysiak et al. 2018). Moreover, reduction of *Pacs-2* levels also results in improved insulin sensitivity and improved liver steatosis in ob/ob mice (Arruda et al. 2014). In addition, a recent study from our laboratory found that PACS-2 was mainly expressed in renal tubules and was decreased in kidney of STZ-induced diabetic mice and patients with DKD (Li et al. 2022). But the effects of PACS-2 on renal lipid metabolism and lipid-related kidney injury of DKD remain unclear.

In this study, we demonstrated that tubule-specific deletion of *Pacs-2* gene in high fat diet/streptozotocin (HFD/STZ)-induced diabetic mice aggravated lipid accumulation and lipid related kidney injury. The precise mechanisms involve the promotion of lipid synthesis and inhibition of cholesterol efflux by inducing the expression of sterol *O*-acyltransferase 1 (SOAT1) and then activating its downstream sterol regulatory element-binding proteins (SREBPs).

## Materials and methods

### Animal experiments and procedures

Proximal tubule-specific *Pacs-2* knockout (*PT-Pacs-2*^−/−^) mice were generated by the Cre-LoxP recombination strategy as previously described (Li et al. 2022). Cre-negative littermate wild-type mice (*Pacs-2*^fl/fl^) were used as controls. *PT-Pacs-2*^−/−^ mice and their control group *Pacs-2*^fl/fl^ mice were fed with a high-fat diet (HFD) (45% fat, ResearchDiets, USA) for 1 month at 4 weeks of age, followed by intraperitoneal injection of 100 mg/kg streptozotocin (STZ) (Sigma-Aldrich, USA). After the injection, mice with random blood glucose levels > 12 mmol/l were included for the experiment. They were named diabetic *PT*-*Pacs-2*^−/−^ mice and diabetic *Pacs-2*^fl/fl^ mice, respectively. Then the mice were maintained for another 20 weeks of HFD feeding. Mice fed with a standard fat diet (SFD) were used as non-diabetic controls, called control *Pacs-2*^fl/fl^ mice and control *PT*-*Pacs-2*^−/−^ mice, respectively. The mice were randomly assigned to four groups. At the end of the last week, mice were sacrificed and anesthetized with sodium pentobarbital. Urine and kidney tissues were collected for various experiments. The sample size was estimated from the effect of the study. All the experiments were approved by the Medical Ethics Committee of Central South University and followed the NIH guidelines for the care and use of laboratory animals.

### Assessment of physiological features and renal function

Body weight and blood glucose were measured every 2 weeks. Urine albumin was measured by a Mouse MAU (Microalbuminuria) ELISA Kit (Sangon Biotech, China) according to the specification of this kit. Urine creatinine levels were measured in the same samples using CicaLiquid-N CRE (Kanto Chemical Co Inc, Japan). Urine *N*-acetyl-β-d-glucosaminidase (NAG) levels were measured with a NAG assay kit (Jiancheng Bioengineering, China) following the manufacturer’s protocol.

### Morphological analysis

Kidney tissues from mice were fixed with 4% paraformaldehyde (PFA) for 14–16 h, embedded in paraffin, sectioned, and stained with hematoxylin–eosin (HE), periodic acid Schiff (PAS), Masson staining, and observed under light microscopy. Tubular damage and glomerular damage were assessed using a semiquantitative scoring system, as described previously (Sun et al. 2011).

### Transcriptome analysis

The transcriptome analysis was conducted by Seqhealth Technology Co., LTD (Wuhan, China). Total RNA from the renal cortex of control *Pacs-2*^fl/fl^ mice and control *PT-Pacs-2*^−/−^ mice was extracted using RNAiso Plus (TaKaRa, Japan) according to the manufacturer’s instructions. The RNA concentration was determined by Nanodrop 2000 spectrophotometer (Thermo Fisher Scientific, USA) and the integrity was tested by 1.0% agarose gel electrophoresis. 5 μg total RNAs were used for mRNA sequencing library preparation. After the library quality inspection is qualified, ~ 300 bp products were sequenced in HiSeq X10 system (Illumina, San Diego, CA, USA). For the RNA-seq data analysis, the data was aligned to the mouse reference genome to obtain comprehensive transcript information and differentially expressed genes (DEGs) as described previously (Ma et al. 2021).

### Immunohistochemistry (IHC)

Paraffin-embedded renal sections were deparaffined in xylene, rehydrated in ethanol and antigen repaired in citrate buffer. After blocking endogenous peroxidase activity with peroxidase blocking solution, the sections were exposed to 5% bovine serum albumin (BSA) and sequentially incubated with primary antibody, anti-Adipophilin (1:200, proteintech, 15294-1-AP, China) overnight at 4 ℃. After washing, the sections were incubated with Goat Anti-Rabbit secondary antibody (1:200, Servicebio, G1213, China), treated with diaminobenzidine, and counterstained with hematoxylin. Finally the sections were dehydrated, soaked in xylene, air-dried, and sealed. Ten images under brightfield were randomly taken for per section in a blinded fashion. Images were obtained using a Nikon microscope and analyzed with Image-Pro plus 6.0.

### Immunofluorescence (IF)

Paraffin-embedded renal sections were blocked in 5% BSA followed by primary antibody, Anti-SOAT1 (1:200, abclonal, A6311, China), incubation overnight at 4 ℃. After washing with Phosphate-buffered saline (PBS), sections were incubated with Alexa Fluor-488-conjugated goat anti-rabbit antibody (1:1000, abcam, ab150077, USA) at 37 °C for 1 h. The nuclei were stained with 4ʹ,6-diamidino-2-phenylindole (DAPI) (SouthernBiotech, China). Five images were randomly taken for each group in a blind fashion. Images were obtained using a Nikon microscope and analyzed with ImageJ software as described previously (Gao et al. 2020).

#### Oil Red O staining

Oil Red O staining was performed in a common manner as previously described (Pei et al. 2016; Yuan et al. 2017). Briefly, the mouse kidney tissues were fixed in 4% PFA for 24–48 h and was dehydrated in 30% sucrose solution overnight. Then the tissues were embedded in O.C.T. compound and cryocut cross-sections (5 mm) were prepared. The frozen sections were air-dried at room temperature and then stained with Oil Red O dye (Servicebio, China) according to the manufacturer’s instructions. HK-2 cells were fixed in 4% PFA for 30 min. After washing with PBS, HK-2 cells were stained with Oil Red O dye for 30 min and then dipped into 60% isopropanol three times. The deposition of lipid droplets in tissue or HK-2 cells was observed under a Nikon microscope. Images were obtained in a blinded manner and analyzed with ImageJ software.

### Cell culture and treatments

The human proximal tubular cell line (HK-2 cells) was obtained from ATCC and cultured in Dulbecco’s Modified Eagle’s Medium (DMEM)/F12 supplemented with 10% FBS and 100 U/mL penicillin plus 0.1 mg/mL streptomycin. One day before transfection, cells were grown to ~ 70% confluence. Then HK-2 cells were transfected with siRNAs or plasmids by Lipofectamine 3000 (Invitrogen, USA) in accordance with the manufacturer’s instructions. pcDNA3 hPACS-2 Flag plasmid was a gift from Professor Gary Thomas (Department of Microbiology and Molecular Genetics, University of Pittsburgh, Pittsburgh, USA). PACS-2 siRNA was purchased from RiboBio Co., Ltd (China). SOAT1 siRNA were purchased from Tsingke Biotechnology Co., Ltd (China). Scrambled siRNAs (Tsingke Biotechnology Co., Ltd, China) and empty vector pcDNA 3.1 vector were applied to parallel cultures as negative controls. Subsequently, cells were stimulated with high glucose plus palmitic acid (HGPA; final concentration 30 mmol/L glucose and 300 μmol/L of the saturated free fatty acid palmitate [16:0]) for 24 h then harvested (Wu et al. 2021a; Sun et al. 2021; Lin et al. 2022; Wu et al. 2021b). The control group used mannitol and fatty acid free BSA (Solarbio, China) as the isotonic solvent control group.

### Bodipy staining

HK-2 cells were washed using PBS and stained with BODIPY493/503 (Invitrogen, USA) 1000-fold diluted for 30 min according to the manufacturer’s instructions. Cell nuclei were counterstained hoechst and images acquired by laser confocal microscopy.

### Cholesterol and free fatty acid content determination

Total cholesterol (TC), free cholesterol (FC) and free fatty acid (FFA) content in HK-2 cells was determined using the Micro Total Cholestenone (TC) Content Assay Kit (Solarbio, China), Micro Free Cholestenone (FC) Content Assay Kit (Solarbio, China) and Free fatty acid detection kit (Solarbio, China) respectively, following the manufacturer’s protocol. Amount of cholesteryl ester (CE) were determined by subtracting the amount of free cholesterol from total cholesterol as described previously (Huang et al. 2018).

### Real time qRT-PCR

The total RNA extracted from the renal cortex and cells using RNAiso Plus was reverse transcribed into cDNA using PrimeScriptTM Reagent Kit (TaKaRa, Japan) with Bio-Rad iCycler system (Bio-Rad, Hercules, CA). Real time qRT-PCR was performed using TB GreenTM Premix Ex Taq II reagent (TaKaRa, Japan) with 7300 Real-Time PCR System (Applied Biosystems). The sequences of the primers are listed in Additional file 2: Table S1. The data are presented as fold changes (2^−ΔΔCt^) normalized to β-actin.

### Western blot analysis

Western blot analysis was performed as described previously (Xu et al. 2016). In brief, protein from mice renal cortex as well as cell pellets was extracted using RIPA buffer (CWBIO, China) containing protease and phosphatase inhibitor cocktail (CWBIO, China). BCA protein assay (Thermo Fisher Scientific, USA) was used for the quantification of protein concentrations. Equal amounts of proteins were used in the Western blot analysis. Primary antibodies specific for PACS2 (1:1000, proteintech, 19508-1-AP, China), FN (1:1000, abcam, ab2413, USA), α-SMA (1:3000, abcam, ab32575, USA), SOAT1 (1:1000, abclonal, A6311, China), SREBF1 (1:500, santa cruz, sc-13551, USA), SREBF2 (1:500, santa cruz, sc-13552, USA), Beta Actin (1:5000, proteintech, 60008-1-Ig, China) were used for western blot.

### Statistical analysis

Data are expressed as mean ± SD. Statistical analyses were performed with GraphPad Prism (version 8.0). The values were analyzed by one-way ANOVA followed by post hoc Tukey’s test or two-way ANOVA followed by post hoc Tukey’s test. Significance was defined as *p < 0.05, **p < 0.01, ***p < 0.001.

## Results

### Tubule-specific Pacs-2 deletion aggravates kidney injury in HFD/STZ-induced mice

**Fig. 1:**
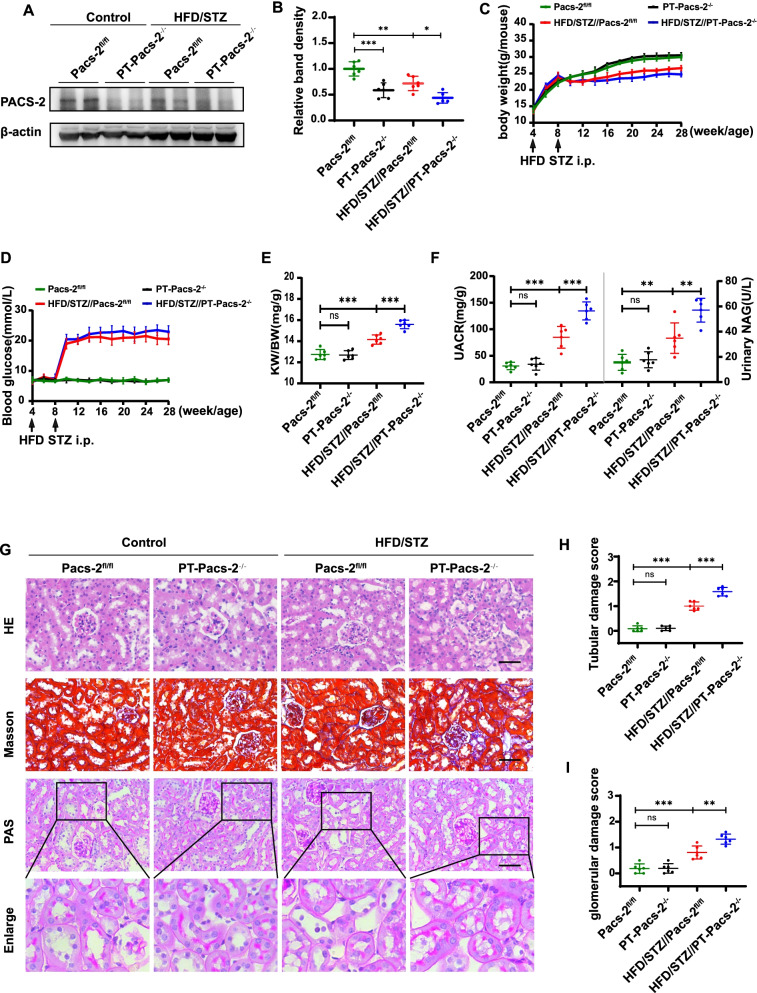
Tubule-specific *Pacs-2* deletion aggravates kidney injury in HFD/STZ treated mice. **A** and **B** Western blot and quantification of PACS-2 expression showing that PACS-2 was knocked out in renal cortex. **C** Body weight and **D** blood glucose are shown. **E** Kidney weight/body weight (KW/BW) ratio, **F** Urine albumin-to-creatinine ratio (UACR) and urinary NAG in mice are shown. **G** Morphological examinations of tubular and glomerular changes by HE, PAS, Masson staining. Scale bars: 50 μm. **H** and **I** Quantification of tubular damage score and glomerular damage score of the kidneys in each group. All data are presented as means ± SD; *p < 0.05, **p < 0.01, ***p < 0.001, ns, not significant. n = 6

The *PT-Pacs-2*^−/−^ mice were generated by a Cre-LoxP recombination system in our group as previously described (Li et al. 2022), which was confirmed by western blot analysis of extracted renal cortex. And there was no significant difference in the expression of PACS-2 in other organs between *Pacs-2*^fl/fl^ mice and *PT-Pacs-2*^−/−^ mice (Additional file 1: Figure S1). Mice were induced to develop DKD with HFD/STZ treatment. The protein expression of *Pacs-2* was lower in diabetic *Pacs-2*^fl/fl^ mice compared to that of control *Pacs-2*^fl/fl^ mice, and this decrease was further aggravated in diabetic *PT-Pacs-2*^−/−^ mice (Fig. 1A, B). In addition, the body weight of diabetic mice was lower than that of non-diabetic mice after the injection of STZ (Fig. 1C), and the opposite pattern was observed for the blood glucose level (Fig. 1D). Besides, diabetic *Pacs-2*^fl/fl^ mice had a higher kidney weight/body weight (KW/BW) ratio than that of control *Pacs-2*^fl/fl^ mice, and the level was further increased in diabetic *PT-Pacs-2*^−/−^ mice (Fig. 1E), indicating the development of more severe hypertrophy in diabetic kidneys when tubular *Pacs-2* was deleted. Diabetic *PT-Pacs-2*^−/−^ mice also showed higher urinary albumin and NAG excretion compared to their controls (Fig. 1F). Moreover, histopathological analysis showed that kidney injury was further aggravated in diabetic *PT-Pacs-2*^−/−^ mice compared with diabetic *Pacs-2*^fl/fl^ mice, including notable tubular epithelial disruption, tubular basement membrane thickening, tubulointerstitial fibrosis, hypertrophy of glomeruli and increased mesangial matrix (Fig. 1G–I). These results indicate that deletion of *Pacs-2* in proximal tubular cells promotes the renal injury and progression of DKD.

### Pacs-2 gene deficiency contributes to lipid accumulation in tubular cells of diabetic mice

**Fig. 2:**
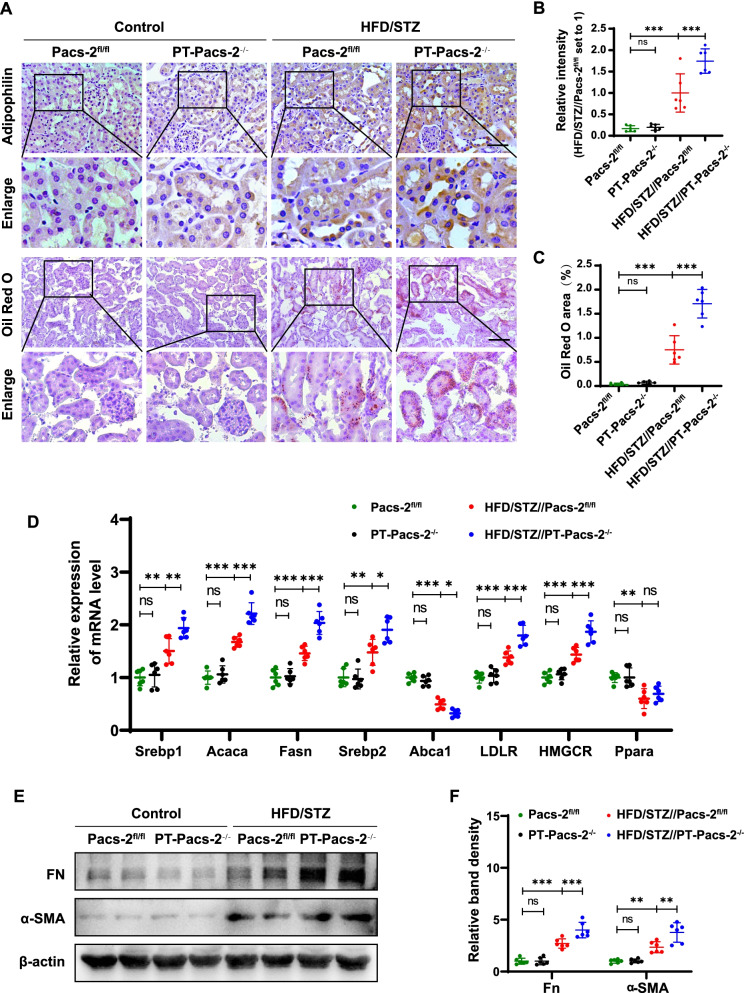
*Pacs-2* gene deficiency contributes to lipid accumulation in tubular cells of diabetic mice. **A** Representative images showing lipid deposition by immunohistochemistry of Adipophilin and Oil Red O staining of kidney from mice. Scale bars: 50 μm. **B** and **C** Relative Adipophilin expression and Oil Red O staining area. **D** Measurement of the mRNA expression of selected markers. **E** and **F** Western blot and quantification of FN and α-SMA. All data are presented as means ± SD; *p < 0.05, **p < 0.01, ***p < 0.001. n = 6

Compared to diabetic *Pacs-2*^fl/fl^ mice, diabetic *PT-Pacs-2*^−/−^ mice had more lipid droplets and neutral lipids deposition in kidney tubules, as assessed by immunohistochemistry of Adipophilin and Oil Red O staining (Fig. 2A–C). Next, we investigated which molecules are associated with perturbed lipid metabolism underlying lipid accumulation in the kidney of diabetic *PT*-*Pacs-2*^−/−^ mice. As shown in Fig. 2D, compared to control *Pacs-2*^fl/fl^ mice, a notable increase of the mRNA levels of *Srebp1* and its downstream acetyl-CoA carboxylase alpha (*Acaca*) and fatty acid synthase (*Fasn*), controlling de novo fatty acid synthesis in the kidney of diabetic *Pacs-2*^fl/fl^ mice were observed, which accompanied with increased expression of *Srebp2* with its downstream low density lipoprotein receptor (*Ldlr*) and 3-hydroxy-3-methylglutaryl-CoA reductase (*Hmgcr*), which mediated cholesterol intake and biosynthesis, respectively, and the levels were further increased in diabetic *PT*-*Pacs-2*^−/−^ mice. In addition, the mRNA level of ATP binding cassette subfamily a member 1 (*Abca1*) controlling cholesterol efflux was decreased in diabetic *Pacs-2*^fl/fl^ mice compared to control *Pacs-2*^fl/fl^ mice, and the levels were further decreased in diabetic *PT-Pacs-2*^−/−^ mice. However, there was no significant difference in the mRNA levels of peroxisome proliferator activated receptor alpha (*Ppara*) between diabetic *Pacs-2*^fl/fl^ mice and diabetic *PT-Pacs-2*^−/−^ mice. Since lipid accumulation was found to accelerate renal tubule fibrosis (Gai et al. 2019), we detected the expression of fibronectin (FN) and smooth muscle alpha-actin (α-SMA). As expect, the expression levels of FN and α-SMA in the renal cortex of diabetic *PT-Pacs-2*^−/−^ mice were higher than those of diabetic *Pacs-2*^fl/fl^ mice (Fig. 2E, F).

### Overexpression of PACS-2 ameliorates lipid accumulation, CE, TC and FFA content increase in HK-2 Cells Induced by HGPA

**Fig. 3:**
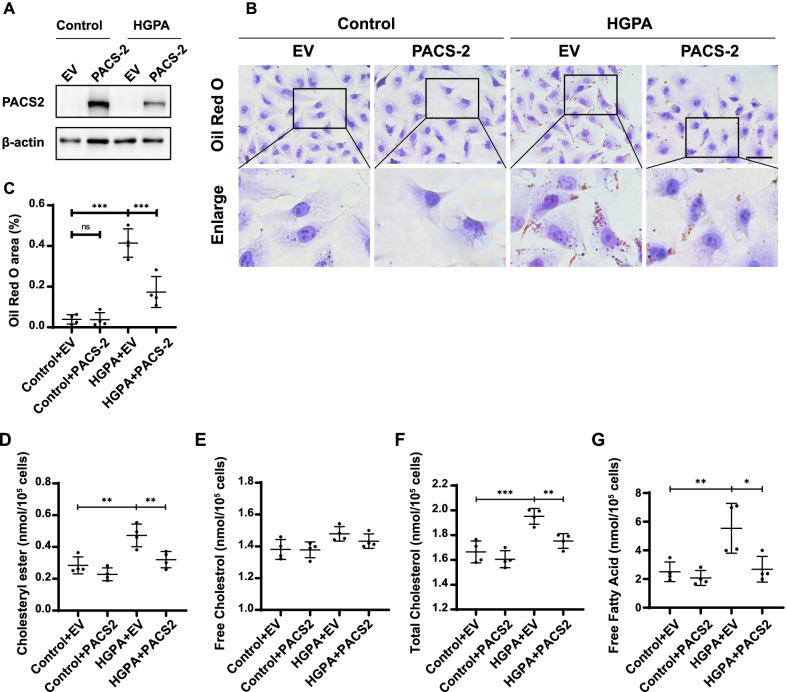
Overexpression of PACS-2 ameliorates lipid accumulation in HK-2 cells induced by HGPA. **A** Gene overexpression efficiency of PACS-2 by western blot analysis in PACS-2 plasmid transfected HK-2 cells. **B** Representative Oil Red O staining images of HK-2 cells in different groups. Scale bar: 20 μm. **C** Oil Red O staining area. **D** Cholesteryl ester content. **E** Free cholesterol content. **F** Total cholesterol content. **G** Free fatty acid content. All data are presented as means ± SD; *p < 0.05, **p < 0.01, ***p < 0.001. n = 4

To confirm the role of PACS-2 in regulation of lipid accumulation, the overexpression of PACS-2 in HK-2 cells was established by transfection with PACS-2 plasmid. A significant increase of PACS-2 expression was observed (Fig. 3A). Overexpression of PACS-2 ameliorated lipid accumulation in HGPA-treated HK-2 cells as determined by Oil red O staining (Fig. 3B, C). As shown in Fig. 3D, F and G, this beneficial effect of regulating lipid metabolism was further verified by the examination of TC, FC and FFA. PACS-2 overexpression resulted in a significant reduction of CE, TC and FFA in HK-2 cells exposed to HGPA ambiance compared to control. However, no change was seen in the FC content (Fig. 3E).

### Identification of SOAT1 as a downstream molecule of PACS-2 and the expression of SOAT1 was increased in the kidney of diabetic mice

**Fig. 4:**
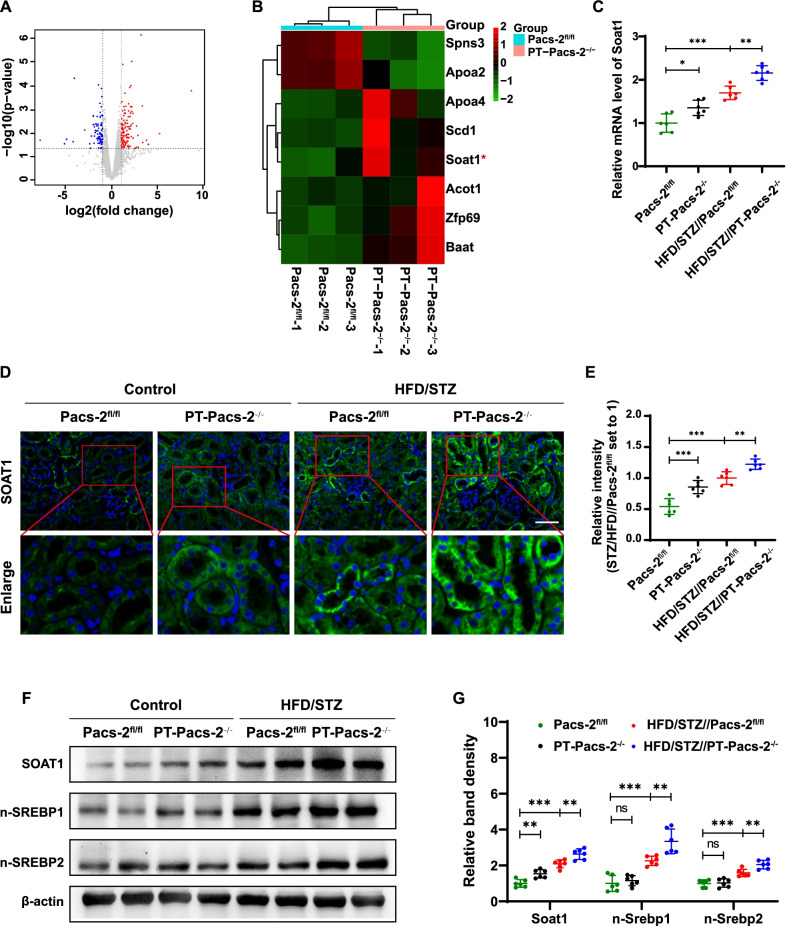
Identification of Soat1 as a downstream molecule of Pacs-2. **A** Volcano plot of DEGs in kidney cortex of control *Pacs-2*^fl/fl^ mice and control *PT-Pacs-2*^−/−^ mice. Splashes represent different genes, and the gray splashes mean genes without significant different expression. The red splashes mean significantly up-regulated genes in control *PT-Pacs-2*^−/−^ mice, and the blue splashes mean significantly down-regulated genes in control *PT-Pacs-2*^−/−^ mice. **B** Heat map of top eight DEGs involved in lipid metabolism; n = 3 mice per group. **C** Real-time qRT-PCR showing the relative mRNA expression of *Soat1*. n = 6 mice per group. **D** Representative immunofluorescence images of SOAT1 (green) in kidney tissues from each group. The nuclei were delineated by DAPI (blue). n = 6 mice per group. Bars = 50 μm. **E** Semiquantification analysis of protein expression. **F** Representative western blot bands of SOAT1, n-SREBP1, n-SREBP2 expression in the kidney cortex, n = 6 mice per group. **G** Relative band density. All data are presented as means ± SD; *p < 0.05, **p < 0.01, ***p < 0.001

Next we explored the mechanism by which PACS-2 regulated lipid metabolism in the diabetic kidney. By transcriptome analysis of renal cortex, 219 differentially expressed genes (DEGs) were identified between renal cortex of control *Pacs-2*^fl/fl^ mice and control *PT-Pacs-2*^−/−^mice, of which 127 genes were up-regulated while 92 genes were down-regulated in control *PT-Pacs-2*^−/−^ mice (Fig. 4A). The heat map of DEGs involved in lipid metabolism showed that *Soat1*, an ER resident protein converting FC to CE, was notably upregulated in control *PT-Pacs-2*^−/−^ mice compared with control *Pacs-2*^fl/fl^ mice (Fig. 4B), and this finding was further confirmed by real time qRT-PCR, immunofluorescence and western blot analysis, respectively (Fig. 4C, D, F and Additional file 1: Figure S2). Immunofluorescence images delineated that the expression of SOAT1 was mainly localized in tubular cells but not in glomeruli. And the increased fluorescence intensity of SOAT1 was observed in diabetic *Pacs-2*^fl/fl^ mice compared to control *Pacs-2*^fl/fl^ mice. Furthermore, *Pacs-2* deletion in tubular cells further increased the expression of SOAT1 (Fig. 4D, E). Similar results were found by western blot analysis (Fig. 4F, G). Besides, by western blot analysis, the intensity of the bands reflected as the expression of N-terminal cleavage product of SREBP1 and SREBP2 (n-SREBP1 and n-SREBP2), which represented the activation of SREBP1 and SREBP2, was notably increased in diabetic *PT-Pacs-2*^−/−^ mice compared to diabetic *Pacs-2*^fl/fl^ mice. This data indicated that deletion of *Pacs-2* may activate SREBP1 and SREBP2, the key transcription factors of lipid metabolism, by increasing the expression of SOAT1 in diabetic conditions.

### Treatment of HK-2 cells with PACS-2 and SOAT1 siRNA regulates the expression of n-SREBPs and lipid accumulation

**Fig. 5:**
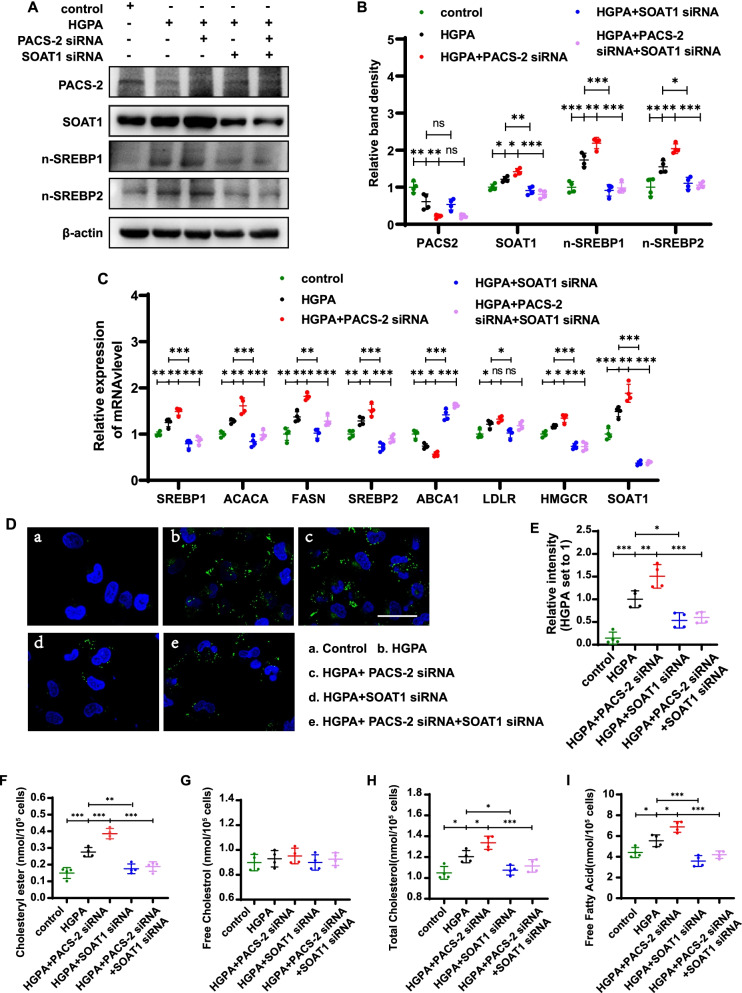
Treatment of HK-2 cells with PACS-2 and SOAT1 siRNA regulates lipid metabolism. **A** and **B** Representative western blot and quantification of PACS-2, SOAT1, n-SREBP1, n-SREBP2 in HK-2 cells. **C** Measurement of the mRNA expression of selected markers. **D** Representative images of cells stained with Bodipy (green); nuclei stained with Hoechst (blue). **E** Quantification of the Bodipy staining. **F** Cholesteryl ester content. **G** Free cholesterol content. **H** Total cholesterol content. **I** Free fatty acid content. All data are presented as means ± SD; *p < 0.05, **p < 0.01, ***p < 0.001. n = 4

Since SOAT1 may play a critical role in the process of PACS-2 modulating the lipid accumulation in tubular cells under DKD condition, next we further detected the effect of silencing PACS-2 and SOAT1 by siRNA treatment in vitro. Results showed that gene silencing of PACS-2 increased expression of SOAT1 and activation of SREBPs in HK-2 cells exposed to HGPA ambiance compared to cells treated with control siRNA. While the effect was blocked partially in HK-2 cells co-transfected with SOAT1 siRNA (Fig. 5A, B). By real time qRT-PCR analysis, a notably increased expression of *SREBP1*, *ACACA*, *FASN*, *SREBP2, HMGCR* and decreased expression of *ABCA1* were found in HK-2 cells treated with HGPA plus PACS-2 siRNA, but the effect was reversed partially by treating with SOAT1 siRNA. In addition, an increased tendency of the mRNA level of *LDLR* was seen, despite no statistical difference (Fig. 5C). Furthermore, silencing of PACS-2 promoted intracellular lipid deposition and increased the levels of CE, TC and FFA in HK-2 cells exposed to HGPA ambiance, however this effect was partially blocked by treatment with SOAT1 siRNA (Fig. 5D–F, H and I). No change was seen in the content of FC (Fig. 5G). These data indicated that reduced PACS-2 could activate SREBPs and promote lipid accumulation by SOAT1 pathway.

## Discussion

This study was designed to explore the role of PACS-2 in lipid-related kidney injury of DKD condition. We found that tubule-specific *Pacs-2* deletion markedly aggravated lipid accumulation in tubular cells by increasing the expression of SOAT1 and then regulating lipid synthesis, uptake and efflux by SOAT1/SREBPs signaling, eventually resulting in worsening albuminuria excretion and kidney injury. These results also highlighted the importance of lipid metabolism abnormality in the pathogenesis of DKD, and PACS-2 may be a potential therapeutic target of DKD.

Recent studies have shown that *Pacs-2* is associated with insulin resistance and liver steatosis in the liver of obese mice through regulating glucose and lipid metabolism (Arruda et al. 2014; Krzysiak et al. 2018). Arruda et al. (2014) found that the expression of PACS-2 was up-regulated in the liver of ob/ob mice and HFD-induced obese mice, and silencing *Pacs-2* improved insulin sensitivity and liver steatosis by decreasing MAM integrity and improving glucose metabolism, rather than regulating genes involved in de novo lipogenesis or cholesterol metabolism. Krzysiak et al. (2018) also suggested that the loss of *Pacs-2* protected HFD-induced obese mice from both hepatic steatosis and whole-body adiposity by elevating liver SIRT1 activity and its downstream fatty acid oxidation genes. Besides, a recent study showed that the expression of PACS-2 decreased in the kidneys of type 1 and type 2 diabetes mice and *Pacs-2* knockout mice with diabetes displayed deterioration of kidney function (Xue et al. 2021). However the role of PACS-2 in tubular cells under diabetic condition remains to be further elucidated. Here, we found that the expression of PACS-2 was decreased in tubular cells of HFD/STZ-induced diabetic mice, which accompanied with renal injury (Fig. 1). Furthermore tubule-specific *Pacs-2* deletion contributed to lipid accumulation in tubular cells under diabetic condition by increasing FFA and cholesterol synthesis, cholesterol uptake and decreasing cholesterol efflux (Fig. 2). In contrast, overexpression of PACS-2 in HK-2 cells reduced the lipid accumulation and content of CE, TC and FFA, while gene silencing of PACS-2 by siRNA had the opposite effect (Figs. 3 and 5). As we all know, the triglycerides themselves serve primarily a storage function with toxicity deriving mainly from FFA, FC, CE, ceramides, etc. (Listenberger et al. 2003; Nakamichi et al. 2021; Tang et al. 2010), indicating PACS-2 may have a renoprotective role from lipid-related kidney injury in DKD. Besides, control *PT-Pacs-2*^−/−^ mice did not show renal lipid accumulation and kidney injury, suggesting that the effects of *Pacs-2* knockout could be tolerated or compensated in the non-DKD state. Similar observations were previously noted in the liver where knockout of *Pacs-2* in mice maintained on a control diet did not result in the change of whole-body adiposity, hepatic steatosis and insulin resistance (Krzysiak et al. 2018).

SOAT1 is a key molecule in lipid metabolism which can convert free cholesterol to cholesteryl esters to prevent overaccumulation of free cholesterol at cellular membranes (Chang et al. 2009). Liu et al. (2020) found that *Soat1* deficiency in diabetic mice reduced cholesteryl ester content in kidney cortex and protected from disease progression. Here, by transcriptome analysis we identified the mRNA level of SOAT1 was up-regulated in the kidney of PACS-2 deficiency mice and found the expression of SOAT1 was increased in tubular cells of diabetic kidney which accompanied with the up-regulation of SREBPs (Fig. 4). SREBPs are key transcription factors regulating lipid metabolism (Shimano and Sato 2017). Geng et al. (2016) found that inhibition of SOAT1 suppressed glioblastoma growth via blocking SREBP-1-mediated lipogenesis. The activity of SREBPs is tightly regulated by a negative feedback loop trigged by ER membrane cholesterol (Goldstein et al. 2006). When cholesterol builds up in ER membranes, SREBPs remain bound to membranes of the ER and are therefore inactive. While cells are depleted of sterols, the SREBPs move to the Golgi complex where two proteases release the active portions of the SREBPs (n-SREBPs), which then enter the nucleus and activate transcription of target genes such as *FASN*, *ACACA*, *ABCA1*, et al. (Goldstein et al. 2002). In addition, when ER cholesterol increases, cells can esterify it with fatty acid to form CE and sequestrate them into lipid droplets through the activity of SOAT1 (Chang et al. 2006). That’s probably the reason why the activity of SREBPs remain high in cells even though lipids like cholesterol are also high (Geng et al. 2016). To confirm that PACS-2 regulated lipid-related kidney injury through SOAT1/SREBPs pathway, HK-2 cells were transfected with siRNA and exposed to HGPA. PACS-2 siRNA increased the mRNA levels of *SREBP1*, *ACACA*, *FASN*, *SREBP2*, *HGMCR, SOAT1* and the protein levels of SOAT1, n-SREBP1 and n-SREBP2 as well as lipid droplets. However these changes were partially reversed by cotreatment with SOAT1 siRNA (Fig. 5). These data indicated that PACS-2 protected lipid-related kidney injury in DKD via the PACS-2/SOAT1/SREBPs pathway. However, the mechanism of how PACS-2 regulates the expression of SOAT1 in DKD is unclear. Further studies will be required to advance our understanding of PACS-2 modulation of lipid metabolism in DKD.

## Conclusions

In summary, this study shows that PACS-2 is an important molecular participant in lipid-related kidney injury in DKD. PACS-2 gene deficiency in tubular cells can further aggravate renal injury by upregulating the expression of SOAT1, and then, enhancing the activity of SREBPs, eventually leading to lipid accumulation and lipid related kidney injury. Pharmacological targeting of PACS-2 and its related signaling pathways may provide a novel therapeutical target for the development of DKD.

## Supplementary Information

**Additional file 1: Fig. S1.** The expression of PACS-2 in other organs of two groups of Mice. (A and B) Western blot and quantification of PACS-2 in the heart, skeletal muscle and liver of *Pacs-2*^fl/fl^ mice and *PT-Pacs-2*^-/-^ mice. ns, not significant. n = 4. **Fig. S2.** Gene silencing of PACS-2 increases the expression of SOAT1 in HK-2 cells. (A and B) A representative western blot and quantification of PACS-2 and SOAT1 in HK-2 cells under control environment. *p < 0.05, **p < 0.01. n = 4.

**Additional file 2: Table S1.** The sequences of primer pairs.

## Data Availability

The datasets used and/or analyzed during the current study are available from the corresponding author on reasonable request.

## Abbreviations

PACS-2: Phosphofurin acidic cluster sorting protein 2
HFD: High fat diet
STZ: Streptozotocin
SOAT1: Sterol *O*-acyltransferase 1
HGPA: High glucose plus palmitic acid
SREBPs: Sterol regulatory element-binding protein
DKD: Diabetic kidney disease
ROS: Reactive oxygen species
MAMs: Mitochondria-associated endoplasmic reticulum membranes
FACL4: Long chain fatty acid CoA ligase 4
PSS1: Phosphatidylserine synthase 1
SIRT1: Sirtuin 1
SFD: Standard fat diet
NAG: *N*-Acetyl-β-d-glucosaminidase
DEGs: Differentially expressed genes
HE: Hematoxylin–eosin
PAS: Periodic acid Schiff
BSA: Bovine serum albumin
TC: Total cholesterol
FC: Free cholesterol
FFA: Free fatty acid
CE: Cholesteryl ester
