# Supplementary material for: PACS-2 deficiency in tubular cells aggravates lipid-related kidney injury in diabetic kidney disease
Source: Mol Med. 2022 Sep 23;28:117. doi: 10.1186/s10020-022-00545-x (PMC9502582; doi:10.1186/s10020-022-00545-x)
Supplement: Supplementary file 1 — Additional file 1: Fig. S1. The expression of PACS-2 in other organs of two groups of Mice. (A and B) Western blot and quantification of PACS-2 in the heart, skeletal muscle and liver of Pacs-2fl/fl mice and PT-Pacs-2-/- mice. ns, not significant. n = 4. Fig. S2. Gene silencing of PACS-2 increases the expression of SOAT1 in HK-2 cells. (A and B) A representative western blot and quantification of PACS-2 and SOAT1 in HK-2 cells under control environment. *p < 0.05, **p < 0.01. n = 4. [file 10020_2022_545_MOESM1_ESM.docx]

Additional file 1


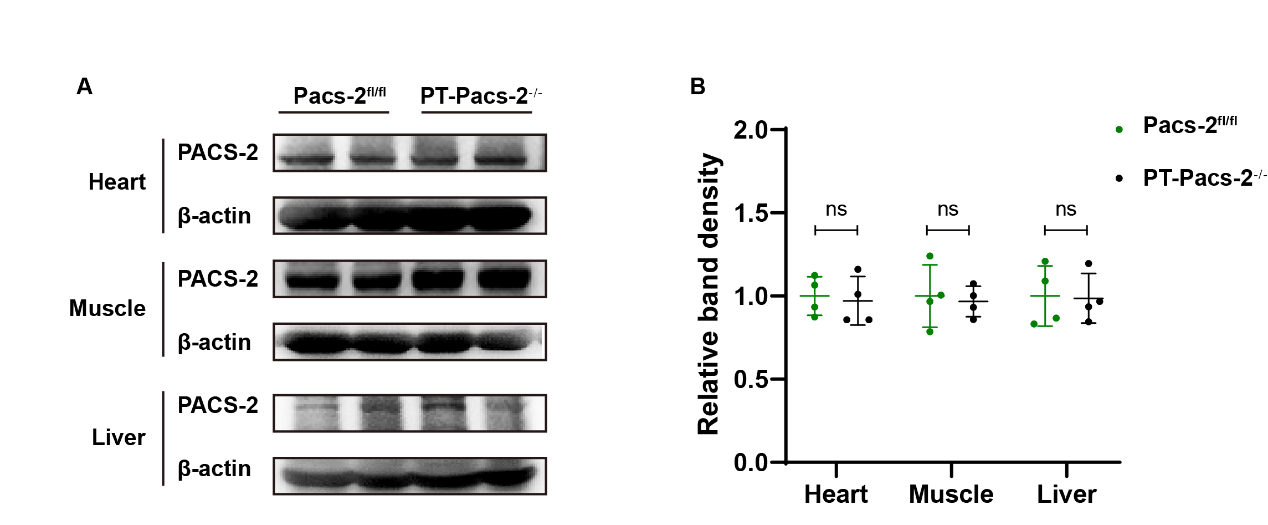


Fig.S1 The expression of PACS-2 in other organs of two groups of Mice. (A and B) Western blot and quantification of PACS-2 in the heart, skeletal muscle and liver of *Pacs-2*^fl/fl^ mice and *PT-Pacs-2^-/-^* mice. ns, not significant. n=4


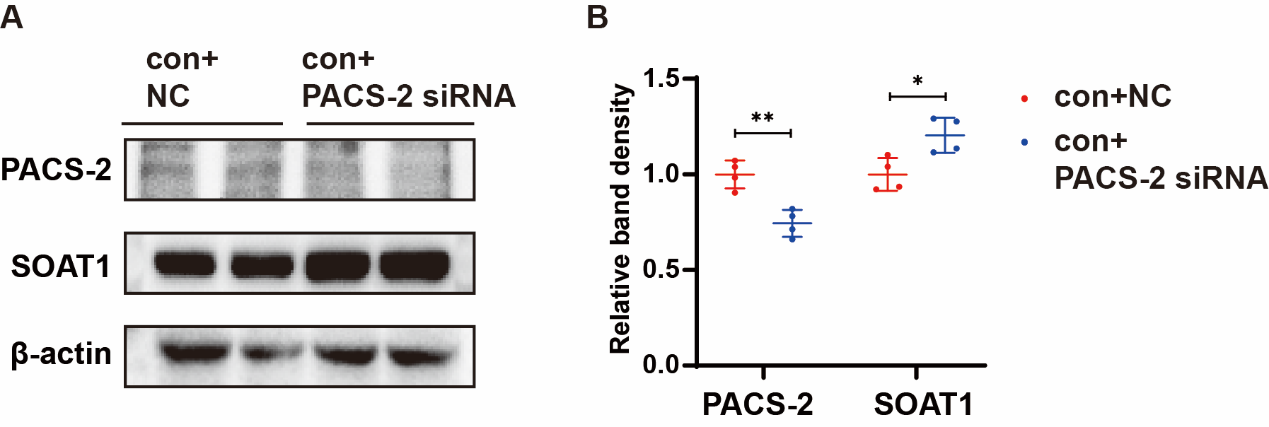


Fig.S2 Gene silencing of PACS-2 increases the expression of SOAT1 in HK-2 cells. (A and B) A representative western blot and quantification of PACS-2 and SOAT1 in HK-2 cells under control environment. *p < 0.05, **p < 0.01. n=4
